# Combining contrast-enhanced ultrasound, CT perfusion and ^99m^Tc-Sestamibi SPECT/CT to guide diagnosis in a case of solid renal tumour

**DOI:** 10.1259/bjrcr.20200115

**Published:** 2020-09-04

**Authors:** Georgios Kalarakis, Katharina Brehmer, Anders Svensson, Rimma Axelsson, Torkel B Brismar, Antonios Tzortzakakis

**Affiliations:** 1 Department of Radiology, Karolinska University Hospital, Huddinge, Stockholm, Sweden; 2 Division of Radiology, Department for Clinical Science, Intervention and Technology (CLINTEC), Karolinska Institutet, Stockholm, Sweden; 3 Medical Radiation Physics and Nuclear Medicine, Functional Unit of Nuclear Medicine, Karolinska University Hospital, Huddinge, Stockholm, Sweden

## Abstract

Definitive, pre-operative differentiation of solid renal lesions by ultrasound, contrast-enhanced multiphasic CT or MRI examinations is often not possible. An increasing amount of literature indicates the added value of ^99m^Tc-Sestamibi SPECT/CT, CT perfusion and contrast-enhanced ultrasound in the pre-operative characterisation of solid renal tumours. This case report presents the diagnostic approach of a solid renal tumour that turned out to be a hybrid oncocytic chromophobe tumour in a patient with Stage 3 renal failure by combining the three aforementioned modern examination techniques.

## Case presentation

A 53-year-old asymptomatic female with a previous history of cardiovascular disease and Stage 3 chronic renal failure was referred to Karolinska University hospital. On a routine abdominal ultrasound examination, a renal lesion was incidentally found. Further imaging was ordered to guide differential diagnosis and clinical management.

## Investigations

**Figure 1.**
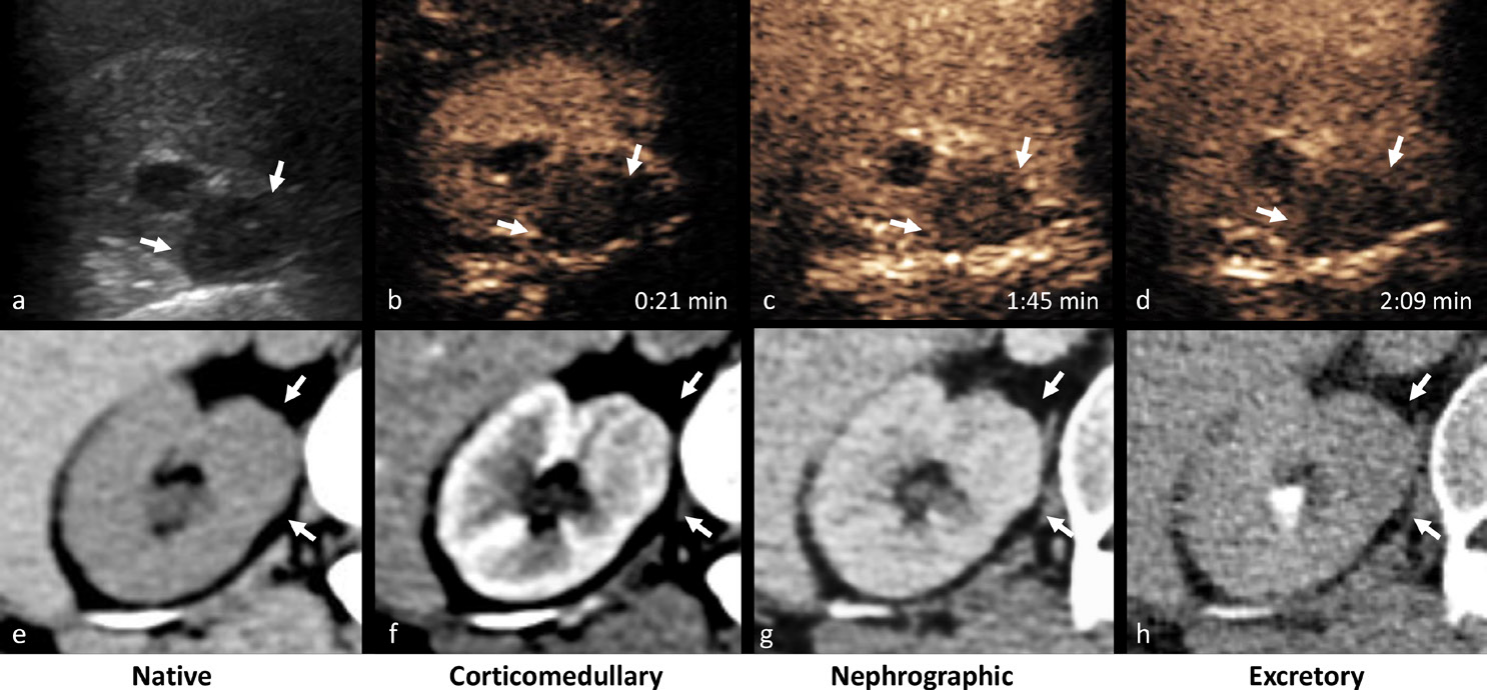
(a) Greyscale ultrasonography shows a hypoechoic cortical lesion in the medial aspect of the right kidney (arrows). CEUS obtained during the cortical (b) and parenchymal (c, d) phase reveals slow enhancement of the lesion which remains hypoenhancing to surrounding normal renal parenchyma. The lesion has the same attenuation as the adjacent renal parenchyma on unenhanced CT (e) and displays progressive enhancement on corticomedullary (f) and nephrographic (g) phase. CEUS, contrast-enhanced ultrasound.

Greyscale ultrasound revealed a hypoechoic lesion in the upper pole of the right kidney measuring 1.5 cm in diameter (Figure 1a). The lesion was avascular on conventional doppler. Contrast-enhanced ultrasound (CEUS) of the kidneys was subsequently performed by injecting a 1.2 ml bolus of contrast agent intravenously (SonoVue, Bracco, Milan, Italy). The lesion showed progressive homogeneous enhancement but remained at all times hypoenhancing to adjacent renal cortex (Figure 1b–d).

**Figure 2.**
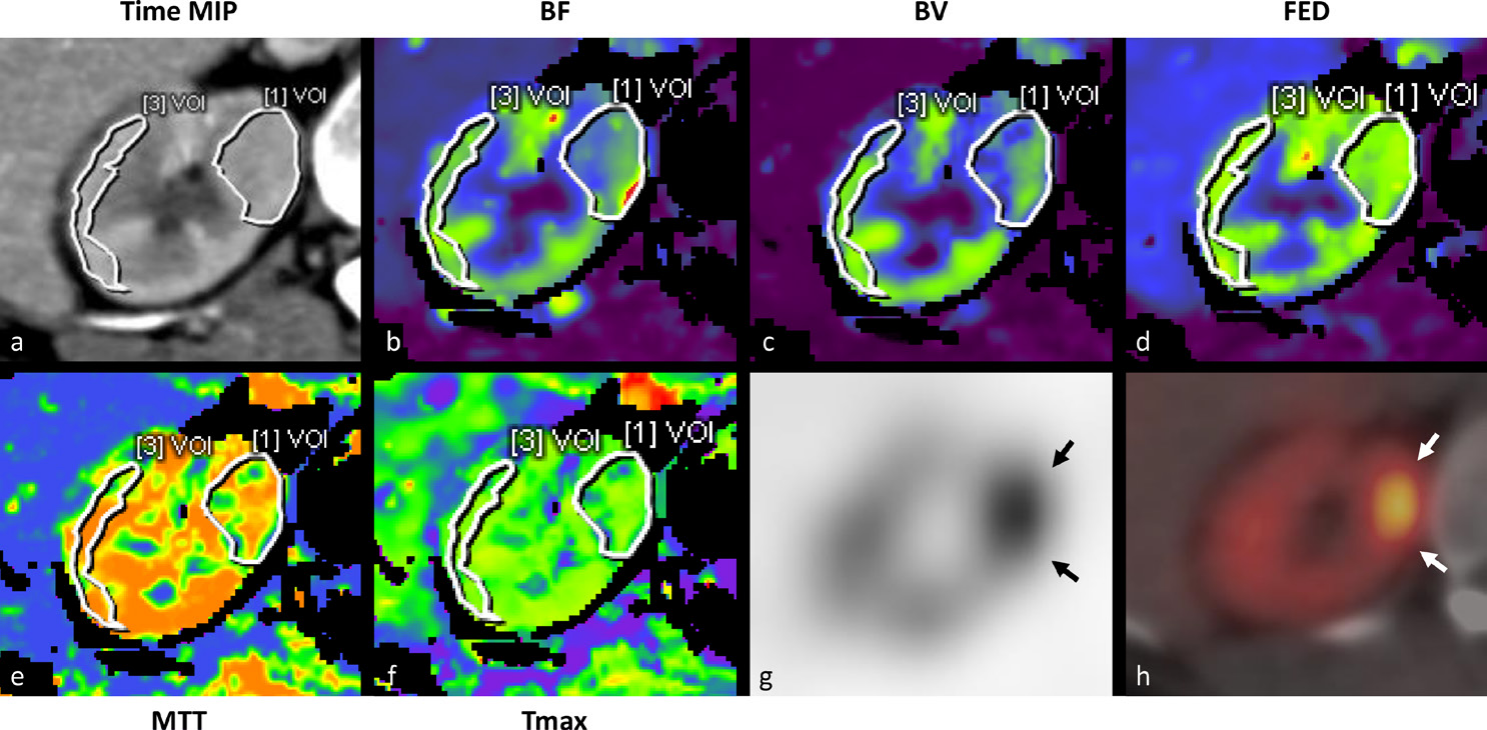
(a–f) Time MIP and parametric maps of BF, BV, FED, MTT, and Tmax of the right kidney. A VOI including the suspect lesion (1) and part of the renal cortex (3) was manually defined in order to calculate the tissue attenuation curve and mean perfusion values. The lesion exhibited lower BF and BV than the renal cortex. On ^99m^Tc-Sestamibi SPECT/CT (g–h), the lesion demonstrated clear focal uptake relative to surrounding renal parenchyma indicating its possible benign character. BF, blood flow; BV, blood volume; FEP, flow extraction product; MIP,maximum intensity projection; MTT, meant transit time; SPECT, single-photon emission computed tomography; Tmax, time to maximum;TTP, time to peak; VOI, volume of interest.

**Figure 3.**
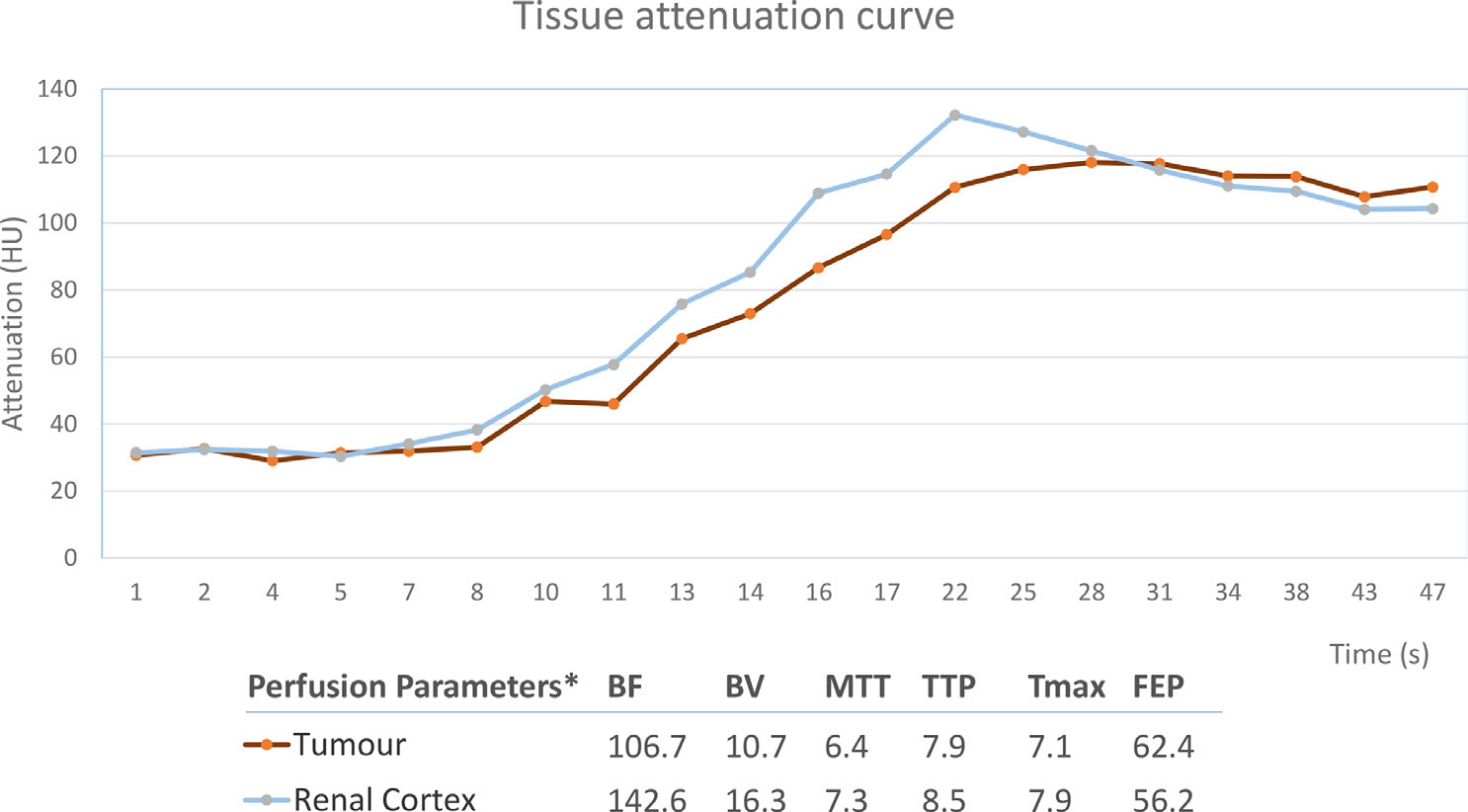
Time–intensity curves and mean perfusion values for the lesion and corresponding renal cortex. The mass was isoattenuating to the renal cortex before contrast arrival and exhibited slow contrast enhancement reaching a maximum of 120 HU. The lesion showed lower BF and BV than the normal cortex, whereas no difference was observed in the rest parametric maps. BF, blood flow (mL/100 g/min); BV, blood volume (mL/100g); FEP, flow extraction product (mL/100 g/min); HU, Hounsfield unit; MTT, mean transit time (s); TTP, time to peak(s); Tmax, time to maximum (s).

To further characterise the solid renal tumour, but still considering the patient’s advanced renal failure, a dynamic perfusion CT study of the kidneys with low intravenous contrast dose was performed. An unenhanced single series of the kidneys was followed by injection of 50 ml of contrast medium (Visipaque 320 mg ml^−1^, GE Healthcare, Chicago, IL) and acquisition of 24 CT volumes for a total duration of 47 s. A conventional scan of the abdomen in the venous and excretory phase was obtained subsequently. Total effective dose for the CT perfusion (CTp) and the following conventional CT study was estimated to be 42.4 mSv. Time average images were reconstructed from the dynamic CT series to generate classic greyscale images in the corticomedullary and nephrographic phases (Figure 1e–h). Perfusion maps of blood flow (BF), blood volume (BV), mean transit time (MTT), time to peak (TTP), time to maximum (Tmax) and flow extraction product (FEP) were generated using dedicated image analysis software (Syngo.Via, Siemens Healthineers Global, Erlangen, Germany) (Figure 2b–f). Perfusion parameters were converted from units of volume to units of mass using a tissue-density conversion factor of 1.05 g ml^−1^. Permeability parameters were not calculated as the acquisition protocol did not include a delayed phase. The tumour and part of the non-tumorous renal cortex were segmented as three-dimensional regions of interest (ROIs). Time–intensity curves and mean perfusion parameters for the lesion and corresponding renal cortex are presented in Figure 3.

The patient underwent a ^99m^Tc-Sestamibi single-photon emission computed tomography (SPECT)/CT examination as a participant in the Molecular Imaging Differentiation of Oncocytomas from Renal cell carcinomas (MIDOR) study. SPECT imaging was performed 60–90 min after the injection of 925 ± 25 MBq^99m^Tc-Sestamibi (produced by the National Centre for Nuclear Research, Poland; distributed by S. Ahlén Medical Nordic AB, Stockholm, Sweden) followed by a CT for attenuation correction and anatomical correlation. Acquired data were reconstructed with HERMES Hybrid Recon^™^ Oncology v. 1.1B (HERMES Medical Solutions AB, Stockholm, Sweden). The renal tumour displayed clear ^99m^Tc-Sestamibi uptake compared to the adjacent renal parenchyma (Figure 2g–h) and thus characterised as photophilic. The effective dose of a ^99m^Tc-Sestamibi SPECT/CT examination was estimated to be around 9,5 mSv.

## Differential diagnosis

The differential diagnosis of a hypoechoic renal lesion without a doppler signal on ultrasound includes simple and complex cysts as well as tumours and pseudo-tumours.^1^ In our case, the lesion demonstrated contrast enhancement on CEUS, effectively excluding the possibility of a blood or debris containing cyst and confirming the solid nature of the finding. Renal pseudo tumours such as a prominent column of Bertin or areas of cortical scarring with compensatory hypertrophy of adjacent parenchyma can mimic a renal tumour on ultrasound. These lesions tend to demonstrate the same pattern and timing of contrast enhancement as normal parenchyma on CEUS. This was excluded in our case, as the lesion was hypoenhancing to adjacent parenchyma on all phases of CEUS. Besides, the patient had no signs of infection, so the presence of kidney abscess or pyelonephritis were excluded as well.

Characterisation of a small solid renal tumour on CT is challenging and often impossible, as both benign solid renal masses (like lipid-poor angiomyolipomas and oncocytomas) and renal cell carcinomas (RCCs) share overlapping imaging characteristics.^2^ In our case, the tumour showed slow progressive enhancement (Figures 1 and 3) that is common both for benign and malignant tumours such as chromophobe (chRCC) and papillary RCCs (pRCC). In contrast, clear cell RCCs (ccRCC) tend to demonstrate avid heterogeneous enhancement, early in the corticomedullary phase.

Regarding perfusion parameters, mean BF and BV of the examined tumour were found to be lower by 25.2 and 34.1% respectively compared to the renal cortex (Figure 3). Only small differences were observed for the other parametric values. Perfusion parameters in our study were compared to those reported in the literature^3–7^ and summarised in Table 1. Absolute BF for the examined tumour was found to be close to the mean value reported for chRCC. Absolute MTT for the examined tumour was closer to the mean reported for RCC rather than for oncocytomas. Renal cortex perfusion values were lower than those reported, possibly reflecting the patient’s reduced GFR.^8^ Due to differences in the analysis method employed by the software used in this study and those reported in the literature, assumptions concerning the nature of the renal tumour based on absolute perfusion values must be viewed with caution.

**Table 1.** Mean perfusion values for renal cortex and different renal tumours pooled from previous studies on CT kidney perfusion

	Author	Software	Renal cortex	Oncocytoma	Minimal fat AML	ccRCC	pRCC	chRCC
**BF** (**mL/min/100 g**)	Wang Y. 2019^3^	GE Perfusion 3.0	-	-	-	214.3	-	-
	Mazzei F. 2014^4^	GE Perfusion 3.0	434.2	477	-	302.87 (all malignant)		
	Chen Y. 2010^5^	GE Perfusion 3.0	454.3	-	-	279.6	52	158.5
	Reiner C. 2013^6^	Syngo Via	-	-	-	230.5^a^	-	-
	Chen C. 2014^7^	Vitrea fx 6.0	305.4	-	191.7	235.2	74.5	120.6
**BV** (**mL/100g**)	Wang Y. 2019^3^	GE Perfusion 3.0	-	-	-	17	-	-
	Mazzei F. 2014^4^	GE Perfusion 3.0	15	18.9	-	15.57 (all malignant)		
	Chen Y. 2010^5^	GE Perfusion 3.0	23.5	-	-	17.97	4.82	14.2
	Reiner C. 2013^6^	Syngo Via	-	-	-	25.8^a^	-	-
	Chen C. 2014^7^	Vitrea fx 6.0	97.8	-	49.3	76.6	28.8	36.2
**MTT** (**s**)	Wang Y. 2019^3^	GE Perfusion 3.0	-	-	-	5.9	-	-
	Mazzei F. 2014^4^	GE Perfusion 3.0	2.72	2.57	-	6.73 (all malignant)		
	Chen Y. 2010^5^	GE Perfusion 3.0	3.62	-	-	6.85	11.74	7.26
**PS mL/min/100 g**	Wang Y. 2019^3^	GE Perfusion 3.0	-	-	-	29.3	-	-
	Mazzei F. 2014^4^	GE Perfusion 3.0	37.74	35.98	-	14.21 (all malignant)		
	Chen Y. 2010^5^	GE Perfusion 3.0	63.95	-	-	25.78	11.9	23.69
	Chen C. 2014^7^	Vitrea fx 6.0	208.4	-	94.1	96.8	58.2	52.5

AML, Angiomyolipoma; BF, Blood Flow; BV, Blood Volume; MTT, Meant Transit Time; PS, Permeability Surface; ccRCC, clear cell RCC; chRCC, chromophobe RCC; pRCC, papillary RCC.

a Reported values converted from units of volume to units of mass using a tissue-density conversion factor of 1.05 g ml^−1.^

Photophilic renal tumours on ^99m^Tc-Sestamibi SPECT/CT examination are of possible benign nature or low malignant potential namely renal oncocytomas, hybrid oncocytic chromophobe tumours (HOCT) or chRCC.^9^

## Management

The case was brought to the multidisciplinary kidney tumour conference. Based on the above-mentioned findings indicating a tumour of benign nature or low malignant potential and due to the patient`s high intraoperative risk, it was decided to perform a percutaneous biopsy of the renal tumour.

## Outcome and follow-up

Routine histopathological staining of the acquired biopsy was initially interpreted as a tumour with morphological characteristics of oncocytoma. Immunohistochemical (IHC) staining of the tissue revealed EMA (+), EpCAM (+), CK7(+), and CD15(-), which were also interpreted as typical for oncocytoma. After review of the biopsy material by two independent pathologists participating in the MIDOR study, consensus was reached and the tissue was reclassified as a HOCT, which is considered to be a subtype of chRCC according to the latest 2016 WHO classification of renal tumours.^10^ HOCT appears to have an indolent clinical course of low metastatic potential although no follow-ups longer than 10 years have been reported in the literature.^11^

Based on the biopsy result and due to the inability of the patient to undergo surgery, the multidisciplinary conference decided to follow-up the patient according to a low-risk cancer protocol (active surveillance). 4 years after the initial diagnosis, the last unenhanced abdominal CT scan and CEUS of the kidneys demonstrated slow growth of the renal tumour from 1.5 to 3 cm in diameter, without evidence of metastasis.

## Discussion

The management of incidentally discovered renal tumours is particularly challenging. Urologists often face the dilemma of opting for an invasive procedure such as partial/radical nephrectomy and thermal ablation or active surveillance.^12^ Although offering a definite treatment, nephrectomy is associated with complications and loss of renal function, which becomes especially relevant for patients with multiple comorbidities and renal failure. Active surveillance entails the risk of delaying intervention for a malignant tumour in the event of misdiagnosis. Therefore, accurate pre-operative characterisation of renal masses is very important. Conventional imaging techniques, namely CT and MRI, often fail to differentiate benign from malignant lesions.^2^ Small renal masses vary widely in their histopathological characteristics and their biological aggressiveness, which complicates their management, even if a biopsy is obtained.^11^ In this context, we explored the added value of emerging imaging methods in the pre-operative characterisation of a solid renal tumour.

Greyscale ultrasound and conventional Doppler is usually the first step in renal lesion assessment. Although simple cysts can be accurately diagnosed on ultrasound, other lesions require a contrast-enhanced multiphase study for further characterisation. CEUS is particularly effective in differentiating a solid renal mass from complex cysts and pseudo-tumours.^1^ In the hands of experienced physicians, CEUS can have excellent sensitivity and good specificity with regard to solid renal tumour characterisation, as shown in a large study by Rübenthaler et al.^13^ However, CEUS is not effective in differentiating oncocytomas and angiomyolipomas from RCCs due to overlapping imaging characteristics.^1,13^ Nevertheless, the lack of nephrotoxicity and the absence of ionising radiation make the use of CEUS important in patients with renal failure or when long-term follow-up is required.^1^

CTp is based on acquisition of multiple scans of the kidneys after contrast media injection. The acquired data set is processed by dedicated software packages to quantify various perfusion parameters. Those parameters are used as indirect signs of tumour angiogenesis, providing important information of the tumour`s ultrastructure. CTp has shown promising results with regard to tumour grading, prognosis and response assessment to antiangiogenic systemic treatment.^14^ Several authors have reported statistically significant differences in perfusion parameters between benign and malignant renal lesions, as well as between different subtypes of RCC (Table 1). However, due to the lack of method standardisation and external validation, its widespread use is limited in everyday clinical practice.

^99m^Tc-Sestamibi is an established mitochondrial radiotracer commonly used in the work-up of primary hyperparathyroidism and for myocardial perfusion imaging. Recent studies have unfolded the ability of ^99m^Tc-Sestamibi SPECT/CT to differentiate benign and indolent renal masses from aggressive renal tumours.^15,16^ On a cellular level, the degree of ^99m^Tc-Sestamibi uptake is considered to reflect the tumour’s mitochondrial content and multi drug resistance (MDR) pump expression.^17^ Benign renal tumours such as oncocytomas, HOCT and some indolent subtypes of chRCCs have high mitochondrial content and relatively low MDR expression, resulting in an avid ^99m^Tc-Sestamibi uptake. On the other hand, the high expression of MDR pumps in malignant tumours, such as ccRCC and pRCC, overweights their mitochondrial content and leads to photopenia. In a meta-analysis of the limited studies available in the literature, Wilson et al^9^ found that ^99m^Tc-Sestamibi SPECT/CT can accurately differentiate benign and low-grade renal lesions from malignant tumours with a sensitivity and specificity that reaches 88 and 95% respectively. Based on these findings, patients with a photophilic renal tumour on ^99m^Tc-Sestamibi SPECT/CT could be considered for percutaneous biopsy and active surveillance instead of surgery. This can result in a significant reduction of operative overtreatment of benign renal tumours, particularly in patients with already limited renal function, as in our case.

## Conclusions

A definitive pre-operative diagnosis of a solid renal tumour can be difficult not only on imaging but also on histopathological grounds. CEUS and CTp cannot characterise renal neoplasia with certainty. ^99m^Tc-Sestamibi SPECT/CT seems to have prominent results in differentiating possible benign from possible malignant renal tumours. In our case, the examined renal tumour was photophilic on ^99m^Tc-Sestamibi SPECT/CT. The biopsy results were suggestive of HOCT and the patient is now followed up according to a local active surveillance programme without evidence of metastasis.

## Learning points

1. A definitive pre-operative diagnosis of a solid renal tumour can be difficult not only on imaging but also on histopathological grounds.
2. CEUS and CTp cannot differentiate benign from malignant renal tumours in a definite way.
3. Photophilic solid renal tumours in ^99m^Tc-Sestamibi SPECT/CT examination have most probably a benign character.
4. There is no evidence of metastatic/aggressive behaviour of HOCT but no reports with a follow up longer than 10 years have been published.
